# An experimental assessment of competitive interactions between sexual and apomictic fern gametophytes using Easy Leaf Area

**DOI:** 10.1002/aps3.11466

**Published:** 2022-04-10

**Authors:** Ondřej Hornych, Lucie Černochová, Aleš Lisner, Libor Ekrt

**Affiliations:** ^1^ Department of Botany, Faculty of Science University of South Bohemia Branišovská 1760, České Budějovice, CZ‐37005 Czech Republic

**Keywords:** apomixis, competition, *Dryopteris*, monoculture, pteridophyte, spore, wood fern

## Abstract

**Premise:**

Few studies have explored competition in fern gametophyte populations. One limiting factor is the tedious measurement of gametophyte size as a proxy for biomass in these small plants. Here, an alternative approach of estimating the number of green pixels from photos was employed to measure the competitive interactions among apomictic and sexual *Dryopteris* gametophytes.

**Methods:**

We cultivated the gametophytes of two apomictic (diploid and triploid) and one sexual (tetraploid) *Dryopteris* species in monocultures and in two‐species mixtures in the ratios 1 : 1 and 1 : 3. The total gametophyte cover of each population originating from 20 spores was assessed using Easy Leaf Area. Assessments were performed weekly between weeks 4 and 10 of cultivation. Additionally, during week 5, the cover of each species in each mixture was estimated separately.

**Results:**

We identified a positive correlation between gametophyte size and ploidy level as well as sexual reproduction. The performance of the tested species in mixtures was dependent on the competitor species identity, indicating the importance of competition between gametophytes.

**Discussion:**

The methods outlined can be used for a rapid assessment of fern gametophyte cover in large gametophyte populations. Ploidy level and reproduction type seem to play a major role in the competitive abilities of fern gametophytes, but more research is needed on this topic.

The fern gametophyte represents a unique organism model that may be utilized for various research purposes. Unlike the gametophytes of other higher plants, fern gametophytes are spatially and nutritionally independent from the sporophyte (Haufler et al., 2016), although they are relatively small (millimeters or centimeters in size). Fern spores, from which gametophytes arise, remain viable for years or even decades in storage (Lloyd and Klekowski, 1970; Windham et al., 1986). They generally germinate within weeks in suitable conditions, and the gametophytes fully develop within months (Lloyd and Klekowski, 1970; Van Nguyen et al., 2020). The shape, size, and sexual expression of fern gametophytes reflect a multitude of abiotic and biotic factors (Korpelainen, 1994; Pajarón et al., 2015; Pangua et al., 2019), which may be studied with a proper cultivation setup (Dyer, 1979).

The presence of fern gametophytes has been demonstrated to affect the size and sexual expression of other gametophytes in populations based on the resulting gametophyte density and the release of antheridiogens into the habitat. When sown at high densities, fern gametophytes tend to be small and male, or may even completely lack gametangia, while at lower densities, gametophytes are larger and female or hermaphroditic (Huang et al., 2004; DeSoto et al., 2008). However, at very low densities or in single‐spore cultures, gametophyte growth may be retarded or abnormal (reviewed by Dyer, 1979). Antheridiogens are pheromones released into the environment by archegoniate (female) gametophytes (possessing a lateral meristem) and absorbed exclusively by undifferentiated asexual gametophytes (Schneller et al., 1990). The receptive gametophytes respond by slowing their growth and producing abundant antheridia (male gametangia) at the expense of archegonia (Schneller, 2008). Most ferns seem to use antheridiogens (Hornych et al., 2021), the effects of which have been observed in natural populations (Tryon and Vitale, 1977). There is an ongoing discussion about whether antheridiogens primarily slow down growth, which prompts antheridial formation, or vice versa (Näf, 1956; Korpelainen, 1994; Quintanilla et al., 2007). Nevertheless, antheridiogens mediate interactions between gametophytes of different developmental stages or ages.

Comparably little is known about the interactions among the gametophytes of multiple species at the same stage/age occurring at densities permitting the formation of both types of gametangia. There are three possible outcomes of such interactions. First, the gametophytes in mixed‐species populations may grow at the same rate as in monoculture. Second, overyielding may occur, leading to gametophytes of at least one species growing faster in the presence of another species, as has been observed in angiosperm sporophytes (Turnbull et al., 2013; Wright et al., 2017). Third, one or more species may underyield (grow smaller) due to competition for resources or chemical allelopathy (Rünk et al., 2004; Testo and Watkins, 2013; Cheng and Cheng, 2015). The effects may be combined; for example, one species may overyield while another underyields in a mixed population. Apart from the negative effects of allelopathy on gametophyte growth (Petersen and Fairbrothers, 1980; Wagner and Long, 1991; Testo et al., 2014), competitive interactions have rarely been studied in fern gametophytes (Testo and Watkins, 2013).

To address this lack of information, we cultivated three fern species of the *Dryopteris filix‐mas* complex (Dryopteridaceae). *Dryopteris filix‐mas* (L.) Schott is a sexually reproducing tetraploid with diploid gametophytes, while *D. affinis* (Newman) Kinahan and *D. borreri* (Newman) Kinahan are apomictic (gametophytes have the same ploidy level as sporophytes). We selected these very closely related species (Fraser‐Jenkins, 2007) to avoid any major differences in developmental patterns; however, their differing ploidy levels (affecting cell size; Robinson et al., 2018; Zhang et al., 2019) and reproductive modes (affecting development speed; Whittier, 1968; Regalado Gabancho et al., 2010; Haufler et al., 2016) may play a role in these interactions.

Fern gametophyte area has been measured for various purposes, as mentioned above. Gametophyte area was previously estimated either indirectly by measuring width and/or length (Tryon and Vitale, 1977; Korpelainen, 1994; Huang et al., 2004) or directly using image processing software (Ganger and Sturey, 2012; Greer et al., 2012; Ganger et al., 2019), such as ImageJ (Quintanilla et al., 2007; DeSoto et al., 2008; Pajarón et al., 2015). Studies have typically measured hundreds or low thousands of gametophytes, and manually outlining each gametophyte individually can be extremely time consuming. A more expedient method would be beneficial for measuring larger quantities of gametophytes, i.e., tens of thousands.

Here, we ask the following questions: (1) Can the sizes of a large number of gametophytes be quickly and reliably assessed through a single analysis of the whole population? (2) Does gametophyte ploidy level and reproduction type affect the absolute and relative gametophyte sizes? (3) Do the gametophytes achieve different sizes in mixed communities and monocultures? (4) Are the relative differences between species consistent during their growth?

## METHODS

### Plants and cultivation conditions used

Fronds of three members of the fern genus *Dryopteris* Adans. (Dryopteridaceae) were collected in 2020 by Libor Ekrt from a garden cultivation (Telč, Czech Republic) and pressed into herbarium vouchers (Table 1). These closely related species (*D. affinis*, *D. borreri*, and *D. filix‐mas*) all belong the *D. filix‐mas* complex. The species range from diploid to tetraploid and reproduce either via apomixis (*D. affinis*: 2*x*, *D. borreri*: 3*x*) or sexually *(D. filix‐mas*: 4*x*). Based on the reproduction type, the spores have the same ploidy level as the sporophytes in the apomicts, but a reduced ploidy level (4*x* ≥ 2*x*) in the sexual species (Table 1).

**Table 1:** Herbarium specimens used for spores in this study (collected from garden cultivation) alongside species details and the place of origin.

Species	Voucher ID	Reproduction type	Origin	Ploidy level (sporophyte/gametophyte)
*Dryopteris affinis* (Newman) Kinahan	CBFS 9795	Apomict	Yorkshire Dales, United Kingdom	2*x*/2*x*
*Dryopteris borreri* (Newman) Kinahan	CBFS 9797	Apomict	Šumava, Czech Republic	3*x*/3*x*
*Dryopteris filix‐mas* (L.) Schott	CBFS 9710	Sexual	Šumava, Czech Republic	4*x*/2*x*

*Note*: CBFS = University of South Bohemia.

For the purpose of cultivation, the wells of ten 12‐well plates were filled to approximately half capacity with 1% agar medium enriched with 25%‐strength Murashige and Skoog inorganic nutrients (Murashige and Skoog, 1962). During the experiment, the plates were kept in a cultivation chamber (MLR‐352 Climatic Test Chamber; PHC Europe B.V., Etten‐Leur, the Netherlands) under a 12‐h light/12‐h dark regime at the lowest light setting (photosynthetic photon flux density: 1700 μmol·m^−2^·s^−1^) and a temperature of 20°C.

### Spore sowing and experimental design

Spores were transferred from dried fern fronds to microscope slides by lightly tapping the fronds. The slide was then observed under a microscope (100× magnification; Olympus CX31; Olympus, Tokyo, Japan) and a total of 20 non‐aborted spores were transferred into each well of the 12‐well plates using tweezers. The spores were attached to the tweezers from the side by static forces; they were not grabbed between the arms of the tweezers as that would have crushed the spores. The presence of the spore in the well was confirmed using a bifocal lens (Olympus S7X7). Sometimes, multiple spores became attached to the tweezers; these were then removed or redistributed evenly within the well. After each spore transfer, the tweezers were cleaned using a paper towel sprayed with ethanol to remove any unwanted spores, debris, or agar pieces. After the spores of one species were sown, the table and equipment were cleaned with ethanol, and the microscope slide was rinsed with water and dried before the next species was used. The plates were covered by a lid and sealed with a double layer of parafilm.

**Figure 1:**
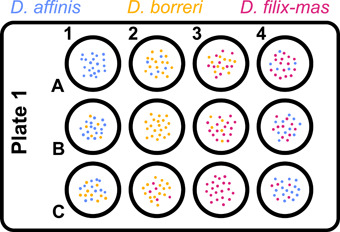
An example experimental setup of the gametophyte competition experiment, showing the *Dryopteris* species sown. The dots within the wells of the plate represent spores and eventually gametophytes, the color of which indicates the species. Each well contains 20 spores of up to two species in the ratios 20 : 0, 15 : 5, and 10 : 10.

The 20 non‐aborted spores in each well were randomly but evenly distributed within the central part of the well (ca. half of the total radius of 1.1 cm). Only the central part of the well was used to eliminate any effect of agar concavity at the edges of the well on the estimate of the gametophyte area. The spores within each well were either from only one species or from two species at a standardized ratio (1 : 1 or 1 : 3). Three species were used, meaning there were 12 possible combinations, each of which was represented once in every plate (Figure 1). To account for the possible influence of the position within the plate, each of the 10 plates had a unique position for every spore combination. Overall, 2400 spores were individually sown during the experiment. Despite the double layer of parafilm, the plates were somewhat ventilated, limiting water condensation. Water droplets have the potential to move spores around when falling onto the agar, which should be considered in alternative setups.

For all wells, an image was taken immediately after sowing. When two species were sown in one well, an additional image was taken before the second species was sown. Therefore, each two‐species well had two images associated with it, one with the spores of one species, and the other with the spores of both species. These two images were combined for an accurate species identification for each spore. During week 5 (see below), the images with identified spores were used to identify the species of the individual gametophytes growing in the wells.

### Observations and data processing

Each well of the ten 12‐well plates was photographed weekly between weeks 4 and 10 using an Infinity 1 camera (Lumenera, Ottawa, Canada) attached to a bifocal lens at 6× magnification; the images were saved as TIFF files. We were unable to record the growth in plates K8, K9, and K10 during weeks 9 and 10 due to pandemic restrictions. Upon preliminary testing, some of the gametophyte area was not green enough to be recognized by Easy Leaf Area (Easlon and Bloom, 2014) (see below); therefore, every image obtained was altered using the Batch Image Manipulation Plugin (BIMP, version 2.5; Francesconi, 2021) for GNU Image Manipulation Program (GIMP, version 2.10; The GIMP Development Team, 2021) using two commands: (a) “change format and compression” → jpeg (necessary for the successful completion of the next step) and (b) “gimp‐drawable‐hue‐saturation” → “hue‐range‐yellow” → “hue offset in degrees” = 10. This process shifts some of the yellow in the gametophyte images to green, making them more suitable for further analyses. The hue offset value was determined based on our preliminary tests. The most appropriate value for this parameter may differ in other setups based on lighting conditions, tested species morphology (e.g., color), or any other relevant factors, and should be calibrated. The calibration process would involve testing the analysis software on unaltered images, observing any potential gametophyte cover not assigned to green pixels, and altering the hue to shift this area into green while preventing any assignment of non‐gametophyte objects as green due to the alteration. One standard alteration setting should be applied to the entire data set unless there are valid reasons for multiple alteration settings being used.

**Figure 2:**
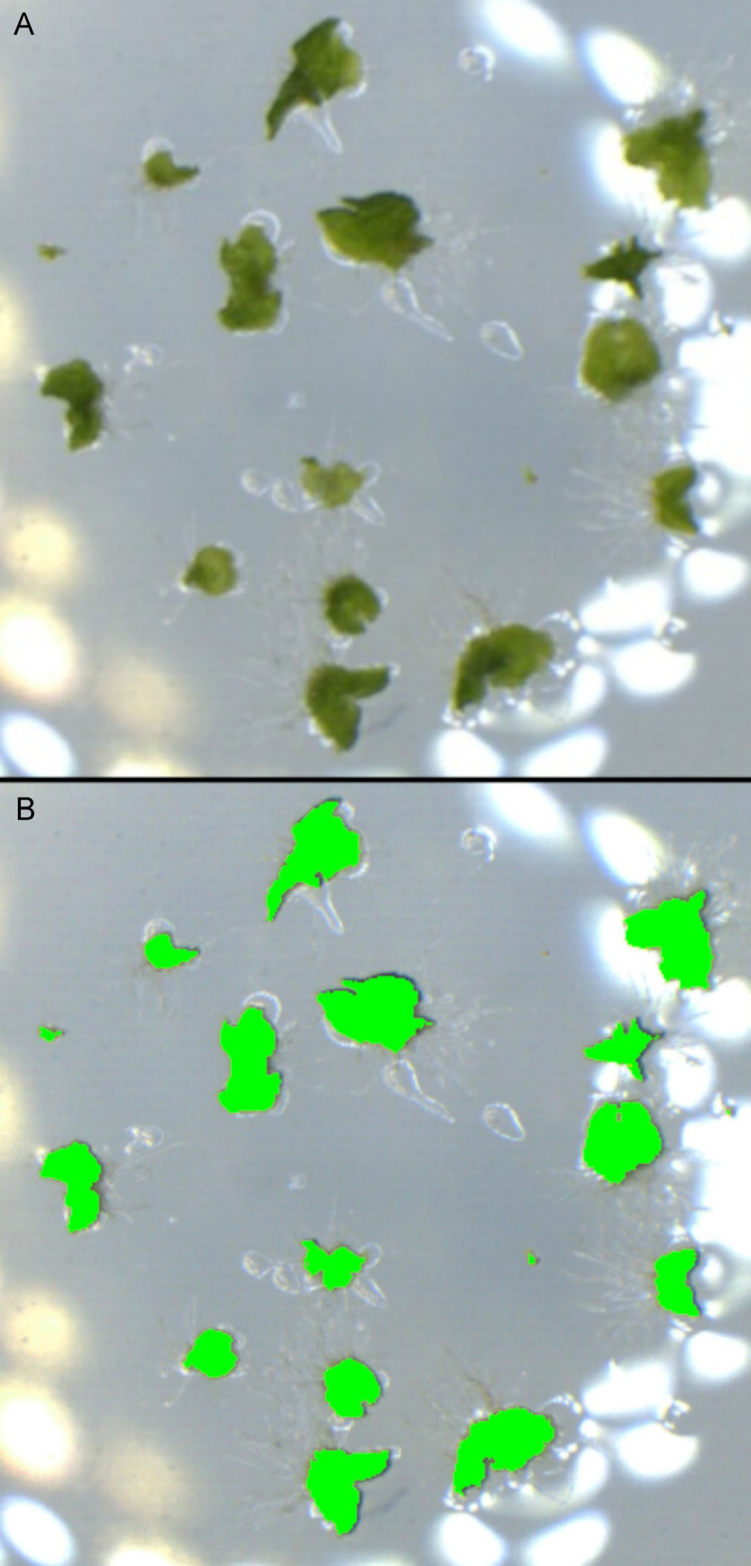
An example image of a gametophyte population (K5–4B) taken during week 5. (A) Image before processing with Easy Leaf Area (but with altered hue to accentuate green pixels). (B) Image processed with Easy Leaf Area, which highlighted and counted the number of green pixels.

The amount of green (in pixels) was obtained for each altered image using Easy Leaf Area. The parameters were set to values that covered most of the gametophyte mass, but did not include any non‐gametophyte parts of the image (for an example, see Figure 2). Easy Leaf Area works with a calibration feature, in which a red object of known area is photographed alongside each sample in one image. The numbers of red and green pixels are then automatically compared to calculate the green area. We were unable to use this feature directly, however. We tried to stick a piece of paper with a 4‐mm^2^ red square under each well, but optical distortions prevented the scanning of the entire red square. Putting the red square directly inside the well disturbed the gametophytes and did not yield accurate results as the squares were randomly tilted due to the concave shape of the agar medium. We therefore photographed ten 100‐mm^2^ red squares separately. The images with red squares were analyzed, and the average number of red pixels was recorded. All images were taken at the lowest possible magnification (6×), making sure that the magnification wheel was moved until it could not go any further. Additionally, the processing speed (parameter in Easy Leaf Area affecting the processed image size) was always set to 2 as this setting alters the number of pixels analyzed.

Week 5 was the last week in which the gametophytes did not physically overlap; therefore, all the images taken at week 5 were processed further: (1) the species of each gametophyte was identified, (2) one species was deleted from the images by manual image alteration, and (3) the altered images were processed as outlined above (BIMP, GIMP, Easy Leaf Area), applying the same settings used for Easy Leaf Area. The new number of pixels was attributed to the non‐deleted species, while the difference between the total pixel number (from the unaltered images) and the number of pixels from the altered images (with one species deleted) was attributed to the deleted species.

The images were taken from above; however, fern gametophytes may grow upward (perpendicular to the surface), especially at later stages of development, meaning the level of perpendicularity affects how the scanned green area reflects the real area of the gametophyte. To address this issue, the analyses outlined below were performed under the assumption that the perpendicularity of the gametophytes is similar at each stage of development, meaning the wells are directly comparable between images taken in the same week (proxy for development stage). Furthermore, fern species may differ in general morphology, which would similarly alter the results, and should be considered.

### Comparison with ImageJ

To compare the accuracy of Easy Leaf Area as a measurement tool for fern gametophytes, 30 images of monocultures from week 5 were also analyzed using ImageJ (Abràmoff et al., 2004), which was used in previous studies. This software calculates the area of a polygon. Each gametophyte in 30 wells was outlined using the polygon selection tool, approximating its shape. To increase the speed and accuracy of the estimates, the polygons were drawn using a Deco 01 (v2; XP‐PEN, Chino Hills, California, USA) graphical tablet. The use of a graphical tablet also increases the level of comfort during the process, but is not necessary. The calibration was performed by outlining the 10 red squares used for the Easy Leaf Area calibration. The total gametophyte area in each well was divided by the average size of 1 mm^2^ based on the calibration. The area estimates in square millimeters were compared between Easy Leaf Area and ImageJ by dividing the cover estimate obtained using the former by the estimate obtained using the latter.

### Statistical analyses

Before the statistical analyses, the data were processed in the following ways: (1) The sizes in pixels were calibrated by dividing the number of pixels by 2190, representing the average number of pixels in 1 mm^2^ based on 10 observations of the 100‐mm^2^ squares. (2) For every well, the theoretical area of each species was calculated by dividing the total area by 20 (total number of sown spores) and then multiplying this number by the number of spores of each species in the well. For the data from week 5, the real area of each species was divided by the theoretically calculated area. This ratio of the real to theoretical area of each species was then standardized to have a mean of 1 by dividing the ratio by its average. (3) Wells with fewer than 20 sown spores (approximately 4% of all wells) were removed from the statistical analyses. The data used for the statistical analyses can be found in Appendix S1.

The abiotic conditions experienced by each gametophyte may be influenced by the position of a well within the plate, specifically the number of bordering sides of each well (e.g., in Figure 1, position 4A has two bordering sides, while position 2B has no bordering sides). This problem should be solved by the experimental design. We confirmed that the number of bordering sides had no significant effect ($X_{2}^{2}$ = 0.604, *P* = 0.739) on gametophyte cover using generalized linear mixed‐effects models (GLMMs) in the *lme4* package (Bates et al., 2015) for R (R Development Core Team, 2020), using a gamma error distribution and logarithmic link function. The identity of each plate was used as a random factor to control for the random variability between the plates, and this approach was used throughout all the following analyses. Based on the non‐significant result, the well position within a plate was not included in further statistical analyses.

For the monocultures, the differences in cover between the individual species, observation period, species reproduction type, and ploidy level were assessed using GLMMs with a gamma error distribution and a logarithmic link function. The analysis was performed in two separate steps, with the first model testing differences in observation period, individual species, and their interaction. The second model quantified the effect of the observation period, species reproduction type, ploidy level, and their mutual interactions. The models cannot be run in a single comprehensive model as species identity strongly overlaps with reproduction type and ploidy level, which does not allow for calculations of their interactions.

Similarly, GLMMs with a gamma error distribution and a logarithmic link function were used to quantify the gametophyte development over time and under different competitive conditions (including monocultures and mixtures), where the species cover was used as a response, and observation period, spore combinations, and their interaction were used as explanatory variables.

To explore how differing competitive environments affect the performance of the constituent species at week 5, the effect of different spore combinations (and thus different species proportions) on a standardized ratio of individual species cover (real to theoretical species cover) was assessed using a linear mixed‐effects model (LMM). Pairwise differences between groups were calculated using the *emmeans* function in the R package *emmeans* (Lenth, 2021) and the *P* value was adjusted using the Tukey method. The analyses were performed separately within each species. Additionally, species monocultures were excluded from the analyses and only differences between mixtures were assessed to avoid circular reasoning because, specifically for week 5, monocultures were used for the calculation of the average individual of each species and this information was used in the assessment of the theoretical area (explained above). Consequently, monocultures also lack any variability, and the ratio always equals 1 (before standardization) because the theoretically calculated area equals the measured gametophyte area.

Please note that in all statistical analyses, a type I (sequential) sum of squares was used. Briefly, each model explains the variability of a response by first using explanatory variable A and then proceeds with explanatory variable B to account for the remaining unexplained variability. In this theoretical example, variable A is effectively a covariate for variable B. The order of the variables in the analyses follows their order in the text, and can also be found in Appendices S2 and S3. The statistical testing in all analyses was performed using a likelihood ratio test.

All statistical analyses were conducted in R software (R Development Core Team, 2020) and visualized in the *ggplot2* package (Wickham, 2016).

## RESULTS

### Monoculture

Of the 2400 spores sown, 2383 germinated, and the growth of these gametophytes was observed over the course of 10 weeks; the remaining 17 spores were missing from five wells. In total, 312.72 cm^2^ of gametophyte area was recorded. The area covered by 20 gametophytes in a single well ranged from 0.30 mm^2^ (Well K2‐1A, week 4) to 209.32 mm^2^ (Well K3‐4B, week 10).

**Figure 3:**
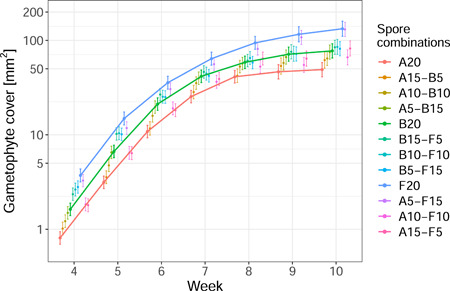
Gametophyte cover as a function of observation period for all 12 combinations of spore mixtures. Error bars indicate the standard error of the mean. For clarity, species monocultures are highlighted by line connections. The letters indicate the *Dryopteris* species used (A = *D. affinis*; B = *D. borreri*; F = *D. filix‐mas*).

The studied species displayed significantly different total coverage ($X_{2}^{2}$ = 86.942, *P* < 0.001) and growth rate (time: $X_{1}^{2}$ = 218.102, *P* < 0.001). The growth rate over time was consistent between the species, as the interaction between these variables was not statistically significant ($X_{2}^{2}$ = 4.000, *P* = 0.129). Overall, the growth of all three species was exponential at first (during weeks 4–7), but then slowed down, reaching a maximum at week 10 (Figure 3). The gametophytes began overlapping by week 6, and perpendicular growth became more prominent over time. The sexual species and the triploid were significantly larger that the apomicts and diploids, respectively (reproduction type: $X_{1}^{2}$ = 56.289, *P* < 0.001; ploidy level: $X_{1}^{2}$ = 30.653, *P* < 0.001). These effects were consistent throughout the observation period (time × reproduction type: $X_{1}^{2}$ = 2.901, *P* = 0.089; time × ploidy level: $X_{1}^{2}$ = 1.198, *P* = 0.274).

### Interactions

The analysis of the entire observation period (all weeks) demonstrated that the total cover in each well is significantly affected by the date of observation (week; $X_{6}^{2}$ = 1283.664, *P* < 0.001) and spore combination ($X_{11}^{2}$ = 332.413, *P* < 0.001; Figure 3). Overall, the spore combinations resulting in the most cover generally included *D. filix‐mas*, while the *D. affinis* monoculture had the least cover. The interaction of week and spore combination was not significant ($X_{66}^{2}$ = 60.769, *P* = 0.659), indicating that the relative differences between groups were consistent throughout the observation period.

**Figure 4:**
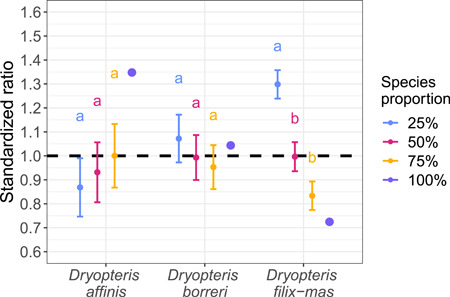
Standardized ratio of the real to theoretical cover for each species and for the three levels of species spore density mixtures (25%, 50%, or 75%) at week 5. For an easier comparison, monocultures (100%, 20 spores total) are passively displayed as points. The monocultures were excluded from the accompanying statistical analysis to avoid circular reasoning because they were used for the calculation of the standardized ratio of the mixtures (see details in the Methods). Each species spore density level sums to 20; the presented value is the number of spores of the targeted species. Error bars indicate standard error of the mean. Different letters denote statistically significant differences between species spore density groups for each species separately.

A detailed analysis in week 5 revealed that the gametophytes of each species frequently under‐ or overperformed compared with the theoretical predictions (Figure 4). After standardization, *D. affinis* generally underperformed when in competition with stronger species (real to theoretical cover at five spores of *D. affinis* was 0.87) and was comparably larger when few (1.00 ratio at 15 spores) or no (1.35 ratio) spores of other species were present. Of the three species, *D. borreri* performed most consistently regardless of the number of spores present (ratios ranging from 0.95 at 15 spores to 1.09 at five spores) and had average growth in monocultures (1.04 ratio). The strongest competitor, *D. filix‐mas*, relatively underperformed in monocultures (0.72 ratio) and fared best when it was rare (1.30 ratio at five spores). The different species competition alternations (species proportions) yielded different performances only when combined with *D. filix‐mas* (*D. affinis*: $X_{2}^{2}$ = 0.569, *P* = 0.753; *D. borreri*: $X_{2}^{2}$ = 1.012, *P* = 0.603; *D. filix‐mas*: $X_{2}^{2}$ = 29.384, *P* < 0.001; Figure 4, Appendix S3). The statistical analyses described above are summarized in Appendices S2 and S3.

### Comparison with ImageJ

In the 30 images analyzed using both software, Easy Leaf Area estimated 86–114% of the gametophyte cover calculated using ImageJ. On average, the estimates by Easy Leaf Area were 3.9% larger. Easy Leaf Area generally overestimated images with larger gametophytes, while Image J overestimated the size of smaller gametophytes.

## DISCUSSION

### Monocultures and competitive interactions

The gametophytes of the three species in our study differed significantly in their growth capabilities, as expressed by their total cover area in monocultures. The diploid gametophytes of the sexual tetraploid *D. filix‐mas* grew largest. In comparison, the apomictic gametophytes were smaller, ordered by ploidy level, i.e., the triploid *D. borreri* was larger than the diploid *D. affinis*. The statistical analysis of the effects of reproductive type and ploidy level was limited by the absence of a hexaploid sexual species (with triploid gametophytes), which is not known in this group; nevertheless, significant differences were found.

Interestingly, the apomicts were smaller than the sexual species overall, which seems to go against the generally presumed faster growth of apomictic gametophytes (Whittier, 1968; Regalado Gabancho et al., 2010; Haufler et al., 2016). One possible explanation is that there may be weaker selective pressure for extensive lateral growth in the apomicts due to the lower requirements for sporophyte formation (no gametangia needed).

Ploidy level was positively correlated with gametophyte size, once reproductive mode was accounted for. This is possibly because fern spore size increases with ploidy level (Barrington et al., 1986, 2020; Ekrt and Koutecký, 2016) and larger spores may carry more resources for gametophyte establishment. Nevertheless, this effect was dwarfed by the differences between apomictic and sexually reproducing ferns in our selected species.

The difference in the gametophyte sizes in the monocultures was correlated with the distribution of the three cultivated species. The largest species, *D. filix‐mas*, has a circumboreal distribution, with considerable coverage outside Europe (Hultén and Fries, 1986). The two apomicts are mostly confined to Europe, with *D. borreri* being more widespread than *D. affinis* (Fraser‐Jenkins, 2007). It is possible that the gametophyte size reflects the competitive ability of a species and, consequently, its abundance and distribution in nature. A similar correlation was found in the *D. carthusiana* (Vill.) H. P. Fuchs complex by Rünk et al. (2004) in Estonia. In their experiment, the three species of this complex differed in their competitive abilities against a grass, *Deschampsia cespitosa* (L.) P. Beauv. These differences reflected the regional species abundance except for *D. dilatata* (Hoffm.) A. Gray, which was competitively strong but comparably rare because it is at its distribution limits in Estonia (Rünk et al., 2004).

Their differing competitive abilities profoundly affected the performance of the tested species in mixed cultivations. One of the predictions stemming from the mass ratio hypothesis is that the dominant species has the most influence on the functioning of communities and the ecosystem (Grime, 1998; Diaz et al., 2007; Pakeman et al., 2011). In our tightly controlled environment (set number of individuals, stable conditions), the number of spores of the dominant species (*D. filix‐mas*) greatly influenced the total productivity of the community, expressed as cover area. The performance of this species was significantly different based on its relative abundance (5, 10, or 15 spores in the mixture; Figure 4). The large size of *D. filix‐mas* gametophytes meant they grew largest when in a minority because they do not suffer from as much strong intraspecific competition. The reverse tendency (stronger interspecific competition) was seen in the weakest competitor, *D. affinis*, which benefited from a lack of stronger competitors and did best in monocultures. The two effects may cancel each other out in *D. borreri*, as this species suffered from the competition of *D. filix‐mas* but performed better when exposed to *D. affinis*. Unfortunately, very few researchers previously focused on the general competitive interactions of ferns at the gametophytic level (Korpelainen, 1997; Testo et al., 2014); thus, our findings provide new insights into these relationships.

### Methodological remarks

We recorded and analyzed the growth of gametophytes originating from 2383 spores using Easy Leaf Area, a fast and convenient tool for assessing the extent of area covered by green tissues. Several modifications from the original usage (Easlon and Bloom, 2014) were necessary. First, the calibration was performed externally due to optical distortions at such a small scale; thus, the level of magnification had to be carefully controlled. The images also had to be altered to shift some of the yellow color into green for the script to be able to detect the entirety of the gametophytes.

Furthermore, a couple of minor methodological issues emerged, of which researchers using this methodology should be aware. A small minority (17 of 2400, 0.7%) of spores were not detected after sowing; they likely did not stick to the agar properly and were blown away. For future studies, the number of spores on each dish should be checked after they have been sealed, and any lost spores should be replaced immediately. Extensive algal growth may also alter the results, and could be reduced by properly sterilizing equipment and work areas, as well as by using freshly prepared sterile plates and media. Nevertheless, if such growth appears, it may be manually deleted from the images (this, however, reduces overall analysis speed), or alternatively the careful choice of settings in Easy Leaf Area should limit or eliminate the inclusion of the algae. If algal growth cannot be sufficiently addressed, the observation should be discarded. This issue is specific to the use of software that analyzes the image as a whole (such as Easy Leaf Area), not one that requires individual gametophytes to be outlined before analysis. It is also important to consider that extensive algal growth might directly affect tested variables, depending on the research topic, i.e., algae could compete with gametophytes for a particular nutrient.

Given that fern gametophyte density affects sexual expression, germination, and growth (Dyer, 1979; Huang et al., 2004; DeSoto et al., 2008), spore sowing density should be chosen based on the research aims. In our study, the spores were sown close enough together to allow any potential interactions to manifest, but far enough apart to provide the gametophytes with space for growth. This trade‐off approach resulted in the overlapping of gametophytes by week 6, in the second half of the experiment. The proper observation of gametophyte size at later stages of development would require lower sowing densities. In such cases, the fact that gametophyte development is abnormal in very low spore densities (reviewed by Dyer, 1979) should be considered.

When sowing fern spores at a given density/number, the issue of spore abortion should also be considered. Most fern species, and especially apomictic ones, abort some of their spores (Wagner and Chen, 1965; Hornych and Ekrt, 2017). Such spores are visibly malformed and will not germinate into gametophytes. If spores are sown individually, aborted spores should be avoided. Alternatively, if the spores are sown as a group, the size of the group should be altered to address the differences in spore abortion (higher abortion = more spore material used).

### Comparison with other methods

Previously used methods of assessing gametophyte area include measuring the width and/or length (Tryon and Vitale, 1977; Korpelainen, 1994; Huang et al., 2004) of the plant. Such methods are expedient, but the results are not comparable for species with diverse gametophyte morphologies, such as is found in *Dryopteris* apomicts (Hornych, personal observation). A more modern approach is to outline and analyze the area of each individual gametophyte (Pajarón et al., 2015; Ganger et al., 2019). A diligent approach using this methodology can yield highly accurate results, but it takes considerably more time than simple two‐dimensional measurements. We would recommend this method for a smaller number of gametophytes (up to hundreds). The methods outlined in the present study can be used to rapidly process a large number of gametophytes (thousands and up). Indeed, in our study, the processing of the 30 images in ImageJ took a similar amount of time as analyzing the entire data set using Easy Leaf Area. We therefore recommend using Easy Leaf Area when large numbers of individuals are used, which in turn balances potential inaccuracies. Nevertheless, the estimates from both Easy Leaf Area and ImageJ are similar. ImageJ overestimates the size of smaller gametophytes, likely due to the difficulty of accurately outlining small objects by hand, which could explain the overall 4% difference in cover to that which was calculated by Easy Leaf Area. A more meticulous approach will yield highly accurate results but take longer to perform.

### Future directions

The outlined methodology could be employed for a variety of research topics. Here, we used these techniques to demonstrate the significance of competitive interactions between fern gametophytes, although further research is needed. The gametophytic phase of ferns is very sensitive to environmental pressures and requires more detailed study. This is especially true for gametophytes in their natural habitat. The total cover of gametophytes in natural populations may be correlated with various ecological factors, and likely differs based on the fern species present. The growth patterns of fern gametophytes may also be affected by their hybrid origin and reproductive mode; however, a proper analysis of these large evolutionary trends would require hundreds of individuals from multiple taxa. A fast and efficient methodology, such as the one reported here, can process large quantities of samples in a relatively short amount of time.

The methodology presented in this study generates results comparable to those generated using more time‐consuming methods. Nevertheless, certain limitations exist. Here we outline how they could be addressed. First, the use of 10 repetitions for each combination and 20 spores per well should theoretically offset possible errors stemming from the improper identification of gametophytes for the analyses at week 5; however, if a lower number of spores/repetitions is to be used, or the accuracy of species identification is critical, molecular barcoding methods may be employed. The identification of fern gametophytes using plastid markers (such as *rbcL*, *matK*, and *trnH‐psbA*) was successfully performed previously, although some related species pairs may be indistinguishable (de Groot et al., 2011; Nitta et al., 2017); therefore, the barcoding method should be tested on the species involved beforehand.

While the studied gametophytes were one cell thick and generally flat, they grew at various angles relative to the agar surface. Easy Leaf Area works with a binary assignment (i.e., either the pixel is green or not); however, some steps may be used to improve the accuracy of the results. One approach would be to alter the hue of images or the setting in Easy Leaf Area to yield pixel counts for various shades of green, which could then be translated into different cell densities; for example, one pixel of “dark green” could be equal to five pixels of “light green.” Alternatively, new scripts could be prepared to specifically analyze the images, taking color value into consideration. Regardless, these methods would have to be carefully calibrated by measuring the dry biomass or the area of flattened gametophytes.

## AUTHOR CONTRIBUTIONS

O.H. and L.E. conceived the study. L.E. collected spore material. O.H. and L.Č. prepared and recorded the cultivations. O.H., L.Č., and A.L. performed the data analysis. O.H and A.L wrote the first draft of the manuscript. All authors contributed to the writing of the manuscript and gave final approval for publication. 

## Supporting information


**Appendix S1**. The data set for the cultivation experiment using three representatives of the genus *Dryopteris*. The theoretical number of pixels was calculated as the total number of pixels divided by the total spore number (usually 20) and multiplied by the number of spores of a given species. The standardized ratio refers to the real : theoretical ratio, standardized to a mean of 1 (monocultures excluded) using division by the mean value.

**Appendix S2**. Results of the generalized linear mixed‐effects models, where fern species cover was used as a response to the observation period and fern species identity in the monocultures; the observation period, reproduction type, and ploidy level in the monocultures; and the observational period and spore combinations. A type I (sequential) sum of squares was used in the analyses.

**Appendix S3**. Results of the linear mixed‐effects models for week 5, in which a standardized ratio of real : theoretical cover for each species was used as a response variable and species spore combinations were used as predictors. A type I (sequential) sum of squares was used in the analyses. The individual contrasts between the three levels of species spore combinations (5, 10, or 15 spores) within each species were quantified using the Tukey post‐hoc method.

## Data Availability

The Supporting Information associated with this article was deposited to Zenodo and may be found online at https://doi.org/10.5281/zenodo.6338274.
